# Assessing the Sustainable Production Potential of Camel Herds in Southern Tunisia

**DOI:** 10.3390/ani16091281

**Published:** 2026-04-22

**Authors:** Chaker Selmi, Mohamed Jaouad, Bernard Faye, Rula Awad

**Affiliations:** 1 Laboratory of Rural Economics and Societies (LR16IRA05), Institute of Arid Regions (IRA), University of Gabès, Medenine 4119, Tunisia; chakerselmi83@gmail.com (C.S.); mjaouad63@gmail.com (M.J.); 2 Center of International Cooperation on Agriculture Research for Development—CIRAD, UMR SELMET, Campus International de Baillarguet, 34398 Montpellier, France; bernard.faye@cirad.fr; 3 Department of Animal Products Marketing, Directorate of Agricultural Marketing and Foreign Trade, Ministry of Agriculture, Amman 11181, Jordan

**Keywords:** camel breeding, arid environments, demographic modelling, DYNMOD, Tunisia, meat and milk production

## Abstract

Camel breeding plays an important role in sustaining pastoral systems in the arid and desert regions of Southern Tunisia, yet the sector remains marginal and insufficiently documented. This study aimed to assess the sustainably production potential of the Tunisian camel herd and to project future meat and milk production. Given the limited availability of detailed statistical data, a demographic simulation model adapted to Tunisian conditions was used to analyze herd dynamics under equilibrium scenarios. Sustainable offtake rates were estimated, and sensitivity analyses were conducted to evaluate the influence of fertility and mortality on productivity. The results indicate that an average annual offtake rate of about 19.1 percent of the herd can be achieved without compromising demographic stability. Productivity gains were primarily driven by improvements in reproductive performance, while further reductions in mortality had a limited effect. Projections for the period 2022 to 2030 show moderate but consistent increases in herd size as well as meat and milk production. These findings suggest that sustainable development of camel production in arid environments relies on adaptive management strategies focused on reproduction, mobility, and market integration rather than conventional intensification.

## Simple Summary

Camels are essential for livelihood in the arid and desert regions of Southern Tunisia, but little information exists on how to use camel herds sustainably without harming their long-term survival. This study aimed to estimate how much meat and milk can be produced while keeping camel populations stable. Using a simulation model adapted to local conditions, we analyzed herd growth, reproduction, and production potential. The results show that about one-fifth of the camel herd can be used each year without reducing herd size. Improving breeding success had a greater impact on productivity than reducing animal deaths, which are already relatively low. Future projections indicate slow but steady increases in herd size and production, highlighting the resilience of camel systems. These findings show that sustainable camel production depends mainly on better reproductive management, continued herd mobility, and improved access to markets, helping support food production and livelihoods in dry regions.

## 1. Introduction

In arid and semi-arid regions (ASAR), rural livelihoods depend on extensive livestock systems that provide food, income, and employment under harsh conditions [1]. These systems face growing pressures from water scarcity and climate variability, intensified by climate change, making their sustainability a global challenge [2]. Among livestock, camels are key components of these systems, capable of thriving in environments where other livestock perform poorly [3]. Consequently, camel production supports food security, income, and risk management for pastoral communities across North Africa, the Middle East, and Asia [3,4]. In Southern Tunisia, camel breeding remains a cornerstone of pastoral land use, supporting livelihoods and enabling the productive use of marginal desert rangelands [5]. Beyond its arid climate, Tunisia represents a transitional context between traditional Saharan pastoral systems and more market-integrated Mediterranean livestock economies [6,7]. Camel production in Southern Tunisia is shaped by the challenges of arid and marginal rangelands while simultaneously adapting to ongoing socio-economic and market changes [5,6]. At the same time, Ongoing processes of semi-sedentarization, rangeland fragmentation, and increasing market integration distinguish Tunisian systems from highly mobile Sahelian herds [5,6,8]. This combination of environmental stress, demographic change, and socio-economic transition makes Tunisia a particularly relevant setting for assessing herd stability and sustainable herd management patterns.

Despite their adaptive advantages, camel production systems are often regarded as weakly productive and economically marginal compared with more intensively managed livestock [4]. This view reflects slow reproductive rates, late age at first calving, long calving intervals, and strong variability in forage availability, which together limit short-term productivity [5]. Consequently, camels are frequently overlooked in livestock development strategies, and their productive potential remains poorly quantified, particularly with respect to sustainable management and long-term herd dynamics [4,9].

In response to these challenges, demographic modelling is a key tool for analyzing livestock herd dynamics under data-scarce conditions, enabling the estimation of sustainable offtake and the identification of key drivers of productivity and resilience [10]. While widely applied to cattle and small ruminants [11], such approaches remain underutilized for camels, despite their central ecological and socio-economic role in arid regions. In Tunisia, camel breeding supports food production, pastoral mobility, and the use of marginal rangelands, yet the sector is poorly documented, constrained by a lack of reliable, disaggregated, and longitudinal data. Demographic modelling therefore provides a robust framework to assess the sustainable production potential of camel herds, essential for designing strategies compatible with the ecological and socio-economic realities of arid pastoral systems.

The present study aims to assess the sustainable production potential of camel herds in Southern Tunisia through a demographic modelling approach. Specifically, the study applies the DYNMOD demographic matrix model to simulate herd dynamics under equilibrium conditions, estimate sustainable offtake rates, analyze the sensitivity of herd performance to key demographic parameters—particularly fertility and mortality—and project herd size as well as meat and milk production over the medium term. By providing a quantitative assessment of camel herd dynamics in an arid context, this work contributes to a broader understanding of how adapted livestock species can support sustainable production, resilience, and food security in dryland ecosystems.

## 2. Materials and Methods

### 2.1. Study Area

**Figure 1:**
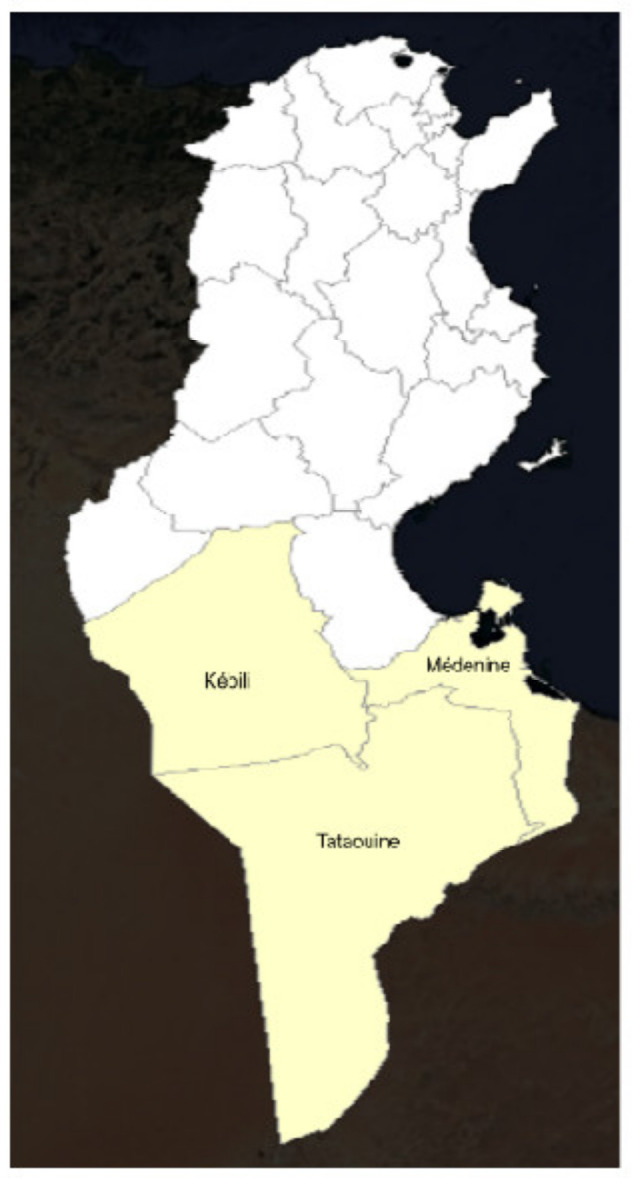
Location of the study area in Southern Tunisia.

The study was conducted in Southern Tunisia, encompassing the governorates of Médenine, Tataouine, and Kebili (32°00′–34°30′ N, 8°30′–11°30′ E; Figure 1). This region belongs to the arid to Saharan bioclimatic zone and is characterized by average annual rainfall below 100 mm, high interannual variability, and frequent drought episodes [12]. Mean annual temperatures exceed 20 °C, with summer maxima often surpassing 45 °C, resulting in chronic water and forage stress [13].

Land use is dominated by extensive rangelands with saline soils and halophytic vegetation [14]. Camel breeding is largely confined to this southern arid belt, where herd distribution is shaped by rangeland availability and transhumant mobility [15,16]. Production systems are predominantly extensive, with herd sizes ranging from 60 to 150 animals, managed individually or collectively and relying mainly on native forage resources. Despite their high adaptive capacity, camels remain underrepresented in national livestock statistics and policy frameworks. In 2022, camel meat accounted for less than 3% of total red meat production, and herd numbers were estimated at approximately 51,850 heads, including about 43,179 females. Cross-border movements since 2011 have introduced additional demographic uncertainty. This combination of environmental exposure, extensive management, and limited institutional visibility makes Southern Tunisia a relevant case for demographic modeling aimed at assessing herd resilience, sustainable herd management, and adaptation pathways.

### 2.2. Demographic Modeling Framework

Demographic dynamics and sustainable production of camel herds were analysed using DYNMOD (dynmod_steady1_v9; https://gitlab.cirad.fr/samir.messad/dynmod, accessed on 2 February 2026), an age–sex structured matrix population model developed by CIRAD and ILRI for demographic projections of livestock populations, and grounded in Leslie–Lefkovitch matrix theory [17]. DYNMOD has been widely applied to evaluate herd growth, productivity, and sustainable offtake in data-scarce pastoral systems [17].

**Figure 2:**
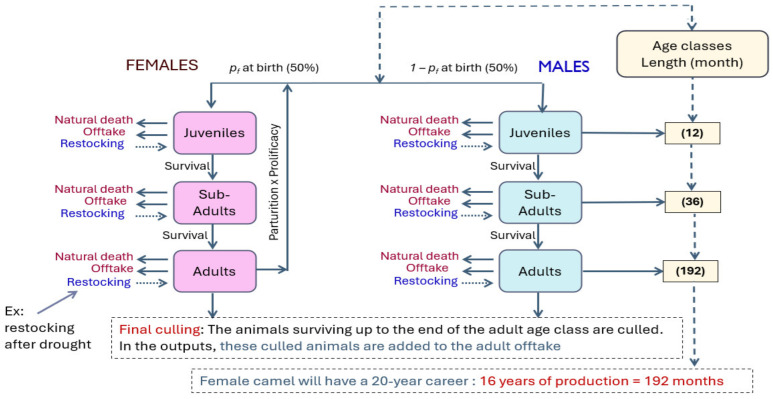
Conceptual structure of the age–sex DYNMOD demographic model for camel herds.

The herd population was disaggregated by sex and grouped into three age classes: juveniles (<1 year), sub-adults (1–4 years), and adults (>4 years). This aggregation reflects common practice in extensive livestock systems where demographic processes are stage-dependent and detailed individual records are unavailable [18]. The conceptual structure of the age–sex demographic model is illustrated in Figure 2.

### 2.3. Model Structure

DYNMOD integrates three main demographic processes: (i) reproduction, defined by age-specific fertility rates of adult females; (ii) mortality, specified for each age–sex class; and (iii) offtake, including sales, slaughter, and transfers. Herd dynamics follow the age–sex transition equation:

(1) $$N_{t+1}=M_{t}\;N_{t}-O_{t}+I_{t}$$

where ($N_{t}$) is the population vector, ($M_{t}$) the transition matrix, and ($O_{t}$) and ($I_{t}$) denote offtake and intake, respectively. Total herd size is obtained by summing across cohorts and follows the aggregate balance:

(2) $$h(t+1)=h(t)+b_{t}-d_{t}-o_{t}+i_{t}$$

The annual change in herd size is given by the difference between inflows (births, animals entering the herd) and outflows (deaths, offtake). Under demographic equilibrium, this change is zero, which defines the maximum sustainable offtake while maintaining a stable herd size and age–sex structure.

### 2.4. Simulation Modes and Temporal Resolution

Two complementary simulation modules were used: STEADY, which estimates the maximum sustainable offtake under demographic equilibrium, defined by constant herd size and stable age–sex structure; PROJ, which simulates herd dynamics over a defined time horizon under non-equilibrium conditions. Simulations were conducted using a monthly time step, with annual outputs obtained by aggregating monthly demographic flows [19].

### 2.5. Model Parameterization and Sensitivity Analysis

Model parameterization relied on demographic rates derived from field surveys and experimental data collected at the Institute of Arid Regions (IRA) of Médenine, complemented by expert knowledge where empirical data were incomplete [16,20]. These parameters represent average demographic conditions under extensive management and exclude major external shocks such as severe droughts or disease outbreaks. The initial population vector corresponded to the observed age–sex structure of camel herds in the study area, with females representing approximately 84% of the total herd, reflecting typical herd management strategies. The average calving rate applied to adult females was set at 0.425 year^−1^, while age-specific natural mortality rates were specified for juveniles, sub-adults, and adults. Live weights and economic values by sex and age class were specified based on local market surveys and expert assessments. The full set of demographics, zootechnical, and economic parameters used in the model is reported in Table 1 and Table 2. Sustainable production was defined as the maximum level of offtake compatible with a stable herd size and age–sex structure, corresponding to a dominant eigenvalue (λ\λ) of the transition matrix equal to unity. Sensitivity analyses were conducted by independently varying age-specific fertility and mortality rates within the observed ranges reported in Table 1 in order to assess their influence on herd growth rate, equilibrium herd structure, and sustainable offtake levels.

### 2.6. Model Validation

To ensure the robustness and reliability of the demographic simulations, both structural and empirical validation procedures were conducted.

- Structural validation

Structural validity was assessed by verifying the internal coherence of the age–sex transition matrix and its consistency with the aggregate demographic balance equation. Under equilibrium conditions (STEADY module), population stability was confirmed by a dominant eigenvalue (λ) equal to unity and by Δh = 0 in the herd balance equation. Iterative projections demonstrated convergence of the age–sex distribution toward a stable structure, confirming that the model produces mathematically consistent demographic trajectories. Because projected quantities of meat, milk, and overall herd size are derived directly from simulated demographic trajectories, the intrinsic population growth rate constitutes a core component of the model. A nominal growth rate of approximately 2.5% per year was applied as a theoretical baseline, reflecting moderate herd expansion under extensive management conditions. This assumption enables the model to estimate future production levels while maintaining realistic demographic dynamics.

- Empirical validation of herd dynamics

The simulated herd structure, characterized by a predominance of adult females (approximately 84% of total herd size), aligns with observed management strategies in extensive camel production systems, where reproductive females constitute the primary productive component of the herd. The magnitude of the simulated herd size is consistent with officially reported numbers in Southern Tunisia, which estimated approximately 51,850 heads in 2022 (Section 2.1). These consistencies support the plausibility of the demographic assumptions used in the model.

- Empirical validation of production outputs

Model-derived meat production levels were compared with official national data. Camel meat accounted for less than 3% of total red meat production in 2022, which provides a general benchmark for evaluating model outputs. Simulated production remains within the range suggested by official statistics, supporting the external validity of the production module.

### 2.7. Parameter Sources and Uncertainty Treatment

The model relies on a deterministic age–sex transition matrix formulated from secondary demographic and production parameters obtained from official national statistics, published studies, and expert-derived reference values tailored to Tunisian camel production systems. There was no primary sampling done for this study.

The model does not depend on sample-based statistical estimation, rendering classical metrics like sample size and confidence intervals inapplicable. Instead, parameter uncertainty was examined via sensitivity analyses on critical demographic variables (fertility and mortality), facilitating the assessment of the stability of equilibrium herd size and sustainable offtake rates amidst plausible biological variability. Future research that includes longitudinal herd-level monitoring data would enable stochastic parameter estimation and probabilistic confidence intervals.

## 3. Results

### 3.1. Production Potential of the Camel Herd

Demographic simulations using the STEADY module of DYNMOD indicate that camel herds in Southern Tunisia can sustain a stable annual offtake rate of 19.1% while maintaining demographic equilibrium (Δh = 0) and a consistent age–sex structure (λ = 1). This deterministic sustainable offtake is derived from the age–sex transition matrix using baseline demographic parameters.

The intrinsic annual growth rate projected under zero-offtake conditions was 2.5%, reflecting the biological growth potential of the herd based on fertility and mortality metrics. This rate corresponds to the dominant eigenvalue (λ > 1) of the transition matrix when there is no offtake. In contrast, the sustainable offtake rate of 19.1% was calculated under stable conditions (λ = 1), where removals are adjusted so that herd size remains constant over time (Δh = 0). Intrinsic growth therefore represents the population’s potential increase, while equilibrium offtake defines the maximum harvest that does not reduce herd size.

Adult females accounted for more than half of the herd, while males represented approximately 16%, consistent with extensive camel systems reported in North Africa. This age–sex structure supports productive capacity, as reproductive females are the primary source of population growth.

Sensitivity analysis conducted on the calving rate (0.40–0.45 year^−1^) revealed a sustainable offtake range of 18.3–19.9%, indicating that fertility is the primary source of uncertainty in the equilibrium offtake. Equivalent proportional reductions in age-specific mortality produced only marginal changes, reinforcing the conclusion that sustainable harvest is largely determined by reproductive performance rather than survival rates.

From a production perspective, the 19.1% sustainable offtake rate represents the biological ceiling for herd offtake without compromising population stability. Meat production is directly proportional to offtake, whereas milk output depends mainly on the number and productivity of lactating females. This rate therefore provides a framework for optimizing herd-derived economic returns: it allows the maximum proportion of animals to be sold while maintaining long-term herd productivity. Exceeding this threshold risks reducing the reproductive base, which would diminish both future meat and milk production and undermine sustainable income generation.

### 3.2. Sensitivity Analysis Under Climatic Variability

Since there are no long-term disaggregated climatic-production datasets, sensitivity ranges were based on the findings of the European project “Promotion of Innovative Camel Systems and Local Camel Value Chains for Sustainable Management of Saharan Territories” (PROCAMED; https://procamed.cirad.fr/en/the-project, accessed on 2 February 2026), which was led by researchers from the livestock laboratory of the Institute for Arid Regions from 2012 to 2015.

During wet years, fertility rates went up by 10–15%, juvenile mortality went down by 10%, and milk yield per lactating female went up by 15%. On the other hand, dry-year conditions were simulated by lowering fertility by 10–15%, raising juvenile mortality by 10–20%, and lowering milk yield by 15–20%, which is what has been seen in arid systems in the field.

The results show that reproductive performance is the main factor that affects both the intrinsic growth rate (λ) and the sustainable offtake. A 10% drop in fertility led to a 3% drop in sustainable offtake, while similar rises in adult mortality had less of an effect. Milk production was very sensitive to changes in the weather in the short term, but herd stability over the long term was more strongly affected by fertility factors than by yearly changes in milk yield.

These results demonstrate that, within camel production systems, demographic resilience is predominantly influenced by reproductive dynamics, whereas production outputs (milk and meat) display greater interannual variability.

### 3.3. Herd Size Projections

Projections generated with the PROJ module indicate a gradual increase in total herd size from 51,850 heads in 2022 to 63,148 heads in 2030 (Table 3), assuming constant demographic parameters and an average annual growth rate of 2.5%. Growth was observed across all age–sex categories, with adult females remaining the largest cohort throughout the projection period. The relative structure of the herd remained stable, indicating demographic consistency over time.

### 3.4. Projected Meat and Milk Production

Based on projected herd dynamics, camel meat production increased from 8760 tons in 2022 to 10,232 tons in 2030 (Table 4). Milk production rose from 14.07 million liters to 17.19 million liters over the same period. Milk production was driven by the increase in the number of adult females, assuming that 42.5% were lactating with an average yield of 515 L per lactating female per year. These projections suggest a steady but moderate increase in camel-derived animal products under current management conditions.

The camel meat production estimates developed here correspond closely to the size of national output of red meats, with camel meat representing the portion of national output of all relevant red meats. This agreement confirms that the production parameters of the model are realistic and appropriately assessed to provide reliable estimates of more expansive management-type production parameters. Despite limited long-term data disaggregated according to demographic growth rates, herd structural characteristics, and actual national herd sizes, evidence of continuing trends in these demographic characteristics supports DYNMOD’s continued application for estimating the sustainable harvesting potential of forage-based production systems under limited data availability.

## 4. Discussion

### 4.1. Camel Herd Dynamics and Demographic Resilience in Arid Systems

The demographic simulations highlight the intrinsic resilience of camel production systems in Southern Tunisia under arid and highly variable climatic conditions. Southern and southeastern Tunisia is among the driest regions of the country, with low and irregular rainfall and substantial inter-annual variability typical of arid climates, where rainfall patterns fluctuate strongly over time and space. Rainfall in the southern arid zone often averages around 150 mm per year with pronounced temporal variability, and recurrent drought episodes are a characteristic feature of the region’s climate, placing chronic environmental stress on grazing resources and livestock systems [13]. In such environments, livestock production systems tend to prioritize long-term stability and persistence rather than short-term productivity maximization. Despite modest reproductive performance, camel herds maintained positive growth trajectories when production remained within biologically sustainable limits, confirming that camels are structurally well adapted to dryland environments with irregular water and forage availability [4,21].

The equilibrium growth rates and stable age–sex compositions estimated in this study align closely with observations from other arid North African systems, highlighting a recurring pattern in extensive camel management [3,22]. In these systems, adult females typically dominate the herd, while male proportions are kept limited—reflecting strategies to maximize reproductive output and buffer against climatic and economic shocks.

Together, these findings emphasize the role of camels as a demographic buffer species. Their ability to sustain positive herd dynamics under extensive management reinforces their strategic importance for climate-resilient livestock production and the livelihoods of pastoral communities facing increasing environmental variability.

### 4.2. Reproductive Performance as the Primary Driver of Productivity

Sensitivity analysis clearly identifies reproductive performance as the dominant driver of herd productivity and production potential. Improvements in calving rate resulted in substantial gains in herd growth and sustainable offtake, whereas further reductions in mortality produced only marginal effects. This outcome reflects the already low baseline mortality of camels and aligns with previous demographic studies using matrix-based models in extensive livestock systems [22].

Reproductive efficiency in camels is strongly influenced by nutritional status, particularly during critical physiological stages such as late gestation and early lactation [23,24]. Targeted nutritional interventions for breeding females therefore represent a high-leverage strategy for enhancing productivity without compromising ecological sustainability. Recent advances in camel reproductive physiology further emphasize the central role of fertility in herd productivity. Camels are induced ovulators, with ovulation triggered by β-nerve growth factor in seminal plasma [25], and they exhibit overlapping follicular waves when ovulation does not occur. Strategies such as ovulation induction or progesterone-based protocols to synchronize follicular waves have been shown to improve breeding management and embryo recovery [26]. In addition, manipulating seasonality through artificial photoperiods or melatonin treatment allows partial control over reproductive activity [25]. These findings indicate that reproductive performance is influenced not only by environmental conditions but can also be enhanced through biotechnological interventions. In the context of our model, improvements in calving rate would directly increase intrinsic herd growth (λ) and expand the sustainable offtake margin, reinforcing the demographic impact of fertility parameters. Importantly, such interventions are compatible with extensive and mobile pastoral systems, provided they are strategically timed and locally adapted.

### 4.3. Herd Offtake Patterns and Sustainability Risks

The estimated sustainable offtake rate of approximately one-fifth of total herd size suggests that camel herds in Southern Tunisia possess a non-negligible productive potential. However, comparisons with observed production practices indicate that this threshold is likely exceeded in some areas, particularly through the slaughter of juvenile females. Although officially prohibited, such practices have been documented in pastoral systems facing market pressures and limited regulatory enforcement [5,16].

Excessive removal of young females reduces the reproductive base of the herd and weakens its demographic buffer against climatic shocks. Similar dynamics have been reported in other arid regions, where short-term income strategies undermine long-term herd viability [20]. These findings underscore the importance of aligning offtake practices with demographic realities to ensure sustainable production.

### 4.4. Herd Growth, Production Potential, and Food Security Implications

Projected increases in herd size, meat output, and milk production suggest that camels can make a meaningful contribution to food systems in arid regions, even under conservative management assumptions. Although absolute production levels remain modest compared with intensive livestock systems, camel products provide a reliable source of animal protein that is less sensitive to climatic shocks [27]. Camel milk is very important for nutrition in dry areas because it gives them a steady source of energy and micronutrients when feed is hard to come by. A recent meta-analysis of 7236 samples found that the average caloric value was 61 Kcal/100 mL, with a range of 55.6–65.5 Kcal/100 mL depending on the season. The highest values were found in winter and the lowest in summer [28]. Its under-representation in official statistics indicates that its contribution to food security and rural incomes is currently underestimated. Strengthening data collection and market recognition of camel products would therefore improve both policy design and resource allocation.

### 4.5. Mobility, Adaptation, and Rangeland Sustainability

The demographic stability observed in the simulations implicitly assumes the continuation of herd mobility, a cornerstone of pastoral adaptation in arid environments. Mobility allows pastoralists to track spatial and temporal variability in forage availability, reducing localized overgrazing and buffering herds against drought-induced feed shortages [15,16].

Any restriction of mobility—through land-use change, administrative barriers, or rangeland fragmentation—would likely increase demographic vulnerability and undermine the sustainability outcomes identified in this study. Maintaining mobility should therefore be recognized not only as a cultural practice but as a functional ecological strategy essential for climate adaptation and rangeland conservation.

### 4.6. Methodological Implications and Limitations

The application of the DYNMOD demographic matrix model demonstrates its suitability for analyzing livestock systems in data-scarce contexts. The modelling framework used in this study shares similarities with the stage-structured camel population model proposed by [29] for feral camels in Australia. Both approaches rely on demographic parameters and deterministic population projections to estimate sustainable removal levels. However, their model explicitly distinguishes between two classes of adult females (with and without calves) in order to account for the long gestation period and extended inter-calving interval characteristic of camels. In contrast, the present model applies an average annual reproduction rate to all adult females, which implicitly integrates the reproductive cycle within a single parameter. Furthermore, whereas the Australian model was developed for a largely unmanaged feral population subject primarily to environmental mortality and culling, the present model represents managed pastoral herds in which demographic structure and offtake are strongly influenced by livestock management practices.

By integrating reproduction, mortality, and offtake rates within a coherent framework, the model provides robust insights into long-term herd dynamics and sustainable offtake. To put the mortality parameters used in the model into context, they were compared to those reported for camel populations in East Africa, where demographic monitoring has been more thoroughly documented. Research from Kenya [30] and Ethiopia [31] shows that calf mortality (0–1 year) is usually between 15% and 30% under normal conditions, but it can go up to 35% during very dry periods. The death rate for adults is usually lower, between 5% and 12%, depending on how much food is available and how often they move around. Even though the climate stress is similar, differences in herd mobility, disease pressure, and conflict-related displacement in some parts of East Africa may make mortality rates more variable than in North African systems. The mortality rates used in this study (4% to 6% for juveniles and 2% to 3% for adults) are in line with what has been seen in East African systems. The relatively low death rates seen in Southern Tunisia are probably due to semi-sedentary management, easier access to veterinarians, and smaller herd sizes compared to transhumant systems in the Horn of Africa. This cross-regional comparison validates the biological feasibility of the demographic parameters employed in the age–sex transition matrix. However, the simulations are based on average demographic conditions and do not explicitly account for extreme climatic events, disease outbreaks, or market shocks. Future research could extend the modeling framework by incorporating stochastic rainfall patterns, drought-induced mortality spikes, and adaptive management responses. Despite these limitations, the results provide a credible baseline for policy analysis and strategic planning.

### 4.7. Implications for Climate-Resilient Livestock Development

The findings support the recognition of camel production systems as a cornerstone of climate-resilient livestock development in ASAR. Rather than pursuing intensification models derived from humid environments, development strategies should build on the camel’s biological adaptations, pastoral mobility, and low-input production logic.

Investments that enhance reproductive efficiency, protect the female breeding stock, and strengthen market integration of camel products are likely to yield durable gains in productivity, resilience, and food security. Demographic modeling tools such as DYNMOD offer valuable decision-support capabilities for evaluating such strategies under current and future climatic uncertainty.

### 4.8. Policy Implications and Recommendations

The findings of this study provide actionable insights for policymakers and development actors seeking to strengthen climate resilience and food security in arid regions through camel breeding systems.

i. Development strategies should explicitly recognize camel breeding as a strategic climate-adapted livestock system. National livestock policies continue to prioritize cattle and small ruminants despite their higher vulnerability to drought. Integrating camels into climate adaptation and food security frameworks would improve alignment between policy objectives and the ecological realities of Southern Tunisia.
ii. Policy interventions should prioritize improvements in reproductive performance through targeted nutritional support for breeding females. Sensitivity analysis indicates that gains in fertility generate substantially higher returns in herd growth and sustainable offtake than further reductions in mortality. Strategic supplementation during critical reproductive stages therefore represents a cost-effective lever for productivity enhancement under extensive management conditions.
iii. Maintaining and facilitating herd mobility should be considered a public good. Securing access to rangelands, regulating transboundary movements through regional cooperation, and adapting land-use policies to pastoral mobility are essential for sustaining herd resilience. Restrictions on mobility would likely increase vulnerability to climatic shocks and exacerbate rangeland degradation.
iv. Strengthening animal health services in remote pastoral areas remains essential for stabilizing herd dynamics and safeguarding production. Preventive veterinary care, disease surveillance, and extension services adapted to pastoral systems can reduce avoidable losses and enhance overall system resilience.
v. improved market integration of camel products is critical for translating biological potential into livelihood and food security gains. Formalizing camel meat and milk value chains, improving processing and quality standards, and supporting local marketing initiatives would enhance income stability for pastoral households while increasing the availability of camel products for consumers.
vi. Demographic models such as DYNMOD should be institutionalized as decision-support tools for livestock planning and policy evaluation. Their capacity to simulate long-term impacts of management and policy choices under climatic and economic uncertainty makes them particularly valuable for evidence-based livestock development strategies.

## 5. Conclusions

This study demonstrates that camel breeding systems in Southern Tunisia constitute a resilient livestock option under arid environmental conditions. Using a demographic matrix modeling approach, we show that camel herds can maintain positive demographic trajectories and stable production levels despite modest reproductive performance and strong climatic constraints. Productivity gains are primarily driven by improvements in reproductive efficiency, particularly through enhanced nutritional management of breeding females, rather than by further reductions in mortality. When offtake remains biologically balanced, moderate but sustained increases in herd size, meat, and milk production can be achieved. These findings confirm the ecological suitability of camel production for arid rangelands and highlight the value of demographic modeling as a robust framework for assessing the long-term sustainability of pastoral systems under increasing climate variability.

## Figures and Tables

**Table 1:** Demographic parameters used in the DYNMOD model for study area camel herds.

Parameters	Sex	Age Class ^1^	Global Herd *	Variation Range (%)
			Structure ^2^ (%)	
**Herd composition**	Females	J	10	8–12
		SA	22	20–24
		A	52	48–56
	Males	J	7	5–9
		SA	5	3–7
		A	4	3–5
**Birth rate**	Females	A	42.5	40–45
**Natural mortality (%)**	Males and Females	J	5	4–6
		SA	4	3–5
		A	2	1–3

^1^ Age classes: J = Juveniles (<1 year), SA = Sub-Adults (1–4 years), A = Adults (>4 years). ^2^ Global Structure: Total herd = 84% females (10% + 22% + 52%), 16% males (7% + 5% + 4%). * Values correspond to average conditions under extensive management.

**Table 2:** Live weight and economic value of camels by sex and age class.

Sex	Age Class ^1^	Live Weight (kg)	Animal Value (TND) ^2^
**Female**	J	100 (95–105)	1500 (1300–1700)
**Female**	SA	300	2500 (2400–2600)
**Female**	A	450	3500 (3300–3700)
**Male**	J	100 (95–105)	1500 (1200–1800)
**Male**	SA	300	3200 (3000–3400)
**Male**	A	575 (550–600)	3500 (3400–3600)

^1^ Age classes as in Table 1. ^2^ Animal value is estimated for local market conditions; ranges indicate observed variation in 2017–2018 surveys. TND = Tunisian Dinar (≈0.35 $).

**Table 3:** Projected evolution of camel herd numbers by age and sex category in Southern Tunisia (2022–2030), generated using the PROJ module of DYNMOD.

	Females				Males				
Year	<1 yr	1–4 yr	>4 yr	Total	<1 yr	1–4 yr	>4 yr	Total	Total Herd
**2022**	5185	11,407	26,962	43,554	3630	2593	2074	8296	51,850
**2023**	5254	11,694	27,670	44,617	3802	2704	2077	8583	53,200
**2024**	5372	11,960	28,387	45,719	3915	2811	2090	8817	54,535
**2025**	5505	12,236	29,113	46,854	4019	2904	2112	9035	55,889
**2026**	5644	12,526	29,850	48,020	4121	2989	2140	9250	57,270
**2027**	5786	12,829	30,601	49,217	4226	3070	2172	9468	58,685
**2028**	5931	13,144	31,369	50,444	4332	3150	2209	9691	60,135
**2029**	6080	13,469	32,153	51,703	4440	3231	2248	9919	61,622
**2030**	6232	13,804	32,957	52,993	4551	3312	2291	10155	63,148

Age classes: <1 yr = Juveniles (<1 year), 1–4 yr = Sub-Adults (1–4 years), >4 yr = Adults (>4 years). Total herd = sum of all sex–age categories.

**Table 4:** Projected camel meat and milk production in Southern Tunisia for the period 2022–2030, based on DYNMOD demographic simulations.

Year	Meat (ton)	Milk (×10^3^ L)	Herd Units (tête)
**2022**	8760	14,070	26,962
**2023**	8636	14,437	27,670
**2024**	8848	14,809	28,387
**2025**	9064	15,186	29,113
**2026**	9286	15,569	29,850
**2027**	9513	15,960	30,601
**2028**	9747	16,360	31,369
**2029**	9986	16,769	32,153
**2030**	10,232	17,188	32,957

Notes: Meat production includes camel carcasses only; official statistics often merge camel and equine meat. Milk production assumes 42.5% of adult females are lactating, averaging 515 L per lactating female per year. Herd units correspond to adult-equivalent animals standardized for economic modeling in DYNMOD.

## Data Availability

No new data were created or analyzed in this study. All parameters used in the demographic model were derived from previously published sources, institutional data, and expert knowledge.

## References

[1] Pankaj P.K., Gaur M.K., Nirmala G., Maruthi V., Pushpanjali, Samuel J., Reddy K.S. (2020). Diversification and land use management practices for food and nutritional security under the climate change scenario in arid and semi-arid regions of India. Food Security and Land Use Change Under Conditions of Climatic Variability: A Multidimensional Perspective.

[2] Al Jassim R., Veerasamy S. (2015). Review paper: Climate change and camel production: Impact and contribution. J. Camelid Sci..

[3] Faraz A., Tauqir N.A., Ishaq H.M., Hussain S.M., Akbar M.A., Mirza R.H., Sufyan A., Rashid S. (2023). Camel Production Profile and Role in Food Security. Int. J. Agric. Biosci..

[4] Amsidder L., Alary V., Duteurtre G., Mnaouer I. (2024). The economic contribution of camel-based livestock systems in North-African drylands: The case of East and South Moroccan provinces. Pastor. Res. Policy Pract..

[5] Rjili H., Muñoz-Ulecia E., Bernués A., Jaouad M., Martin-Collado D. (2023). Evolution of pastoral livestock farming on arid rangelands in the last 15 years. Animal.

[6] Burger P.A., Ciani E., Faye B. (2019). Old World camels in a modern world–a balancing act between conservation and genetic improvement. Anim. Genet..

[7] Alary V., Frija A. (2022). Crop–livestock systems transformation in the semiarid zones of North Africa over a decade: Approach and case-study in Southern Tunisia. J. Agric. Sci..

[8] Ministry of Foreign Affairs of the Netherlands Tunisia Climate Fact Sheet: Arid and Desert Zones of Southern Tunisia [Fact Sheet]. https://prddsgofilestorage.blob.core.windows.net/api/documents/Tunisia_-_Climate_Fact_Sheet/TUNISIA_Climate_Fact_Sheet_EN.pdf?utm_source=chatgpt.com.

[9] Ashour G., Abdel-Rahman S.M. (2022). Camels as a miracle key for animal production sustainability in Egypt. Egypt. J. Anim. Prod..

[10] Pluchinotta I., Zhou K., Zimmermann N. (2024). Dealing with soft variables and data scarcity: Lessons learnt from quantification in a participatory system dynamics modelling process. Syst. Dyn. Rev..

[11] Aragie E.A., Thurlow J. (2022). Modeling the recovery dynamics of Ethiopia cattle population. J. Arid. Environ..

[12] Najjar A., El Mahroug H., Hechlef H., Khaldi S. (2024). Diagnosis of actors and relational flows of camel meat value chain in the Western South of Tunisia. Int. J. Innov. Approaches Agric. Res..

[13] Institut National de la Météorologie (2020). Climat de la Tunisie.

[14] Tlili A., Ghanmi E., Ayeb N., Louhaichi M., Neffati M., Tarhouni M. (2020). Revegetation of marginal saline rangelands of southern Tunisia using pastoral halophytes. Afr. J. Range Forage Sci..

[15] Jemli M.H., Boulajfene H., Azouzi Z., Salem W.B., Khaldi S. (2018). Camel breeding development project in Tunisia. Rev. Mar. Sci. Agron. Vét..

[16] Letaief N., Bedhiaf-Romdhani S. (2022). Camel herd management under pastoral system in southern Tunisia. Demographics of Indigenous Bovine Cattle Farms in Greece, Tunisia and Algeria.

[17] Bahta S.T., Enahoro D.K., Wanyoike F.N., Mensah C., Dizyee K., Rich K.M., Karugia J.T. (2021). Livestock Sector Strategy–Recursive Dynamic Spatial Equilibrium Model (LSS-RDSEM): Model Concept and Description.

[18] Mukhopadhyay S., Piepho H.P., Bhattacharya S., Dublin H.T., Ogutu J.O. (2024). Hierarchical Bayesian integrated modeling of age- and sex-structured wildlife population dynamics. J. Agric. Biol. Environ. Stat..

[19] Bateki C.A., Dickhoefer U. (2020). Evaluation of the modified livestock simulator for stall-fed dairy cattle in the tropics. Animals.

[20] Moritz M., Cross B., Hunter C.E. (2023). Artificial pastoral systems: A review of agent-based modelling studies of pastoral systems. Pastoralism.

[21] Faye B., Konuspayeva G., Magnan C. (2023). Large Camel Farming: A Care-Management Guide from Breeding to Camel Products.

[22] Alzuraiq F., Faye B., Lesnoff M. (2015). Use of demographic model to assess the potential change in camel population and economy: The example of Saudi Arabia. Veterinariya.

[23] Ayyat M.S., Monem U.M.A., Mostafa T.H., Thabet R.M., Abd El-Latif K.M., Al-Sagheer A.A. (2025). Influence of long-term dietary cobalt supplementation on lactation performance and reproductive efficiency in Maghrabi she-camels. Biol. Trace Elem. Res..

[24] Mohammed A.A., Almuyidi A., Almarri H., Alkhalifah H., Alhmad A., Alali H., AlHuwaish O., AlKhawaher M. (2025). Unique characteristics of camel body systems: Adaptation to harsh conditions, productive and reproductive performances: A review. Indian J. Anim. Res..

[25] Arroyo E., Laquiz-Silva N. (2025). Ovarian dynamics and pathological conditions in camelids. Reprod. Domest. Anim..

[26] Waqas M.S., Anouassi A., Tibary A. (2025). Manipulation of ovarian activity in camelids. Clin. Theriogenology.

[27] El Hamrouni A., Khorchani T., Neffati M., Rejeb M.N., Gohis F., Hajji A., Khemiri H., Suissi M., Sghaier Zaafouri M. (2020). Techniques et pratiques de restauration, réhabilitation et systèmes d’exploitation des parcours. Rev. Régions Arid..

[28] Alhaj O.A., Ahmad L., Alenezi A.F., Abodoleh G., Ghazzawi H., Trabelsi K., Bragazzi N.L., Mehta B.M., Faye B., Jahrami H.A. (2023). Does total caloric count of camel milk differ by species, country, season and year of publication: A systematic review, meta-analysis and meta-regression. Int. J. Dairy Technol..

[29] Al-Jassim R., Lisle A. (2016). Prediction and management of feral camel population in Australia. Advances in Conservation Through Sustainable Use of Wildlife: Proceedings of the Conference held in Brisbane, Australia, 30 August−1 September 2016.

[30] Kaufmann B.A. (2000). Camel calf losses and calf care measures in pastoral herds of Northern Kenya: A system view. Rev. Elev. Med. Vet. Pays Trop..

[31] Abdulmaji G., Kumbe A. (2024). A retrospective study on the mortality rate of camel calves, leading causes, and associated risk factors in Borana Zone, Oromia Regional State, Ethiopia. J. Appl. Vet. Sci. Technol..

